# Cognitive outcome is related to functional thalamo-cortical connectivity after paediatric stroke

**DOI:** 10.1093/braincomms/fcac110

**Published:** 2022-04-28

**Authors:** Leonie Steiner, Andrea Federspiel, Nedelina Slavova, Roland Wiest, Sebastian Grunt, Maja Steinlin, Regula Everts

**Affiliations:** 1 Division of Neuropaediatrics, Development and Rehabilitation, Department of Paediatrics, Inselspital, Bern University Hospital, University of Bern, Bern, Switzerland; 2 Graduate School for Health Science, University of Bern, Bern, Switzerland; 3 Psychiatric Neuroimaging Unit, Translational Research Center, University Hospital of Psychiatry and Psychotherapy, University of Bern, Bern, Switzerland; 4 Institute of Diagnostic and Interventional Neuroradiology, Inselspital, Bern University Hospital, and University of Bern, Bern, Switzerland; 5 Department of Diabetes, Endocrinology, Nutritional Medicine and Metabolism, Inselspital, Bern University Hospital and University of Bern, Bern, Switzerland; 6 Pediatric Radiology, University Children's Hospital Basel and University of Basel, Basel, Switzerland

**Keywords:** thalamus, thalamo-cortical connectivity, arterial ischaemic stroke, paediatrics, rs-fMRI

## Abstract

The thalamus has complex connections with the cortex and is involved in various cognitive processes. Despite increasing interest in the thalamus and the underlying thalamo-cortical interaction, little is known about thalamo-cortical connections after paediatric arterial ischaemic stroke. Therefore, the aim of this study was to investigate thalamo-cortical connections and their association with cognitive performance after arterial ischaemic stroke. Twenty patients in the chronic phase after paediatric arterial ischaemic stroke (≥2 years after diagnosis, diagnosed <16 years; aged 5–23 years, mean: 15.1 years) and 20 healthy controls matched for age and sex were examined in a cross-sectional study design. Cognitive performance (selective attention, inhibition, working memory, and cognitive flexibility) was evaluated using standardized neuropsychological tests. Resting-state functional magnetic resonance imaging was used to examine functional thalamo-cortical connectivity. Lesion masks were integrated in the preprocessing pipeline to ensure that structurally damaged voxels did not influence functional connectivity analyses. Cognitive performance (selective attention, inhibition, and working memory) was significantly reduced in patients compared to controls. Network analyses revealed significantly lower thalamo-cortical connectivity for the motor, auditory, visual, default mode network, salience, left/right executive, and dorsal attention network in patients compared with controls. Interestingly, analyses additionally revealed higher thalamo-cortical connectivity in some subdivisions of the thalamus for the default mode network (medial nuclei), motor (lateral nuclei), dorsal attention (anterior nuclei), and the left executive network (posterior nuclei) in patients compared with controls. Increased and decreased thalamo-cortical connectivity strength within the same networks was, however, found in different thalamic subdivisions. Thus, alterations in thalamo-cortical connectivity strength after paediatric stroke seem to point in both directions, with stronger as well as weaker thalamo-cortical connectivity in patients compared with controls. Multivariate linear regression, with lesion size and age as covariates, revealed significant correlations between cognitive performance (selective attention, inhibition, and working memory) and the strength of thalamo-cortical connectivity in the motor, auditory, visual, default mode network, posterior default mode network, salience, left/right executive, and dorsal attention network after childhood stroke. Our data suggest that the interaction between different sub-nuclei of the thalamus and several cortical networks relates to post-stroke cognition. The variability in cognitive outcomes after paediatric stroke might partly be explained by functional thalamo-cortical connectivity strength.

## Teaser

The purpose of the study was to investigate resting-state thalamo-cortical connectivity and its association with cognition in patients after paediatric arterial ischaemic stroke. Steiner *et al.* report that the interaction between different subdivisions of the thalamus and several cortical networks might be essential for post-stroke cognitive performance.

## Graphical Abstract

**Graphical abstract:**
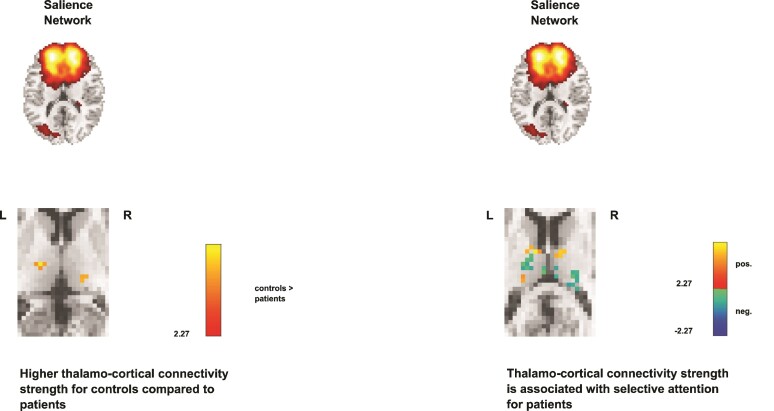

## Introduction

Paediatric arterial ischaemic stroke (AIS) occurs rarely, but two-thirds of survivors may have lifelong neurological, motor, and cognitive deficits,^1–3^ which in turn can lead to reduced health-related quality of life.^4,5^ Post-stroke outcome varies with patient-related factors (e.g. age at stroke and socio-economic status) and stroke- and lesion-related factors, including stroke severity, lesion size, and lesion location.^1,3,6^ However, these factors have only limited predictive power for long-term cognitive outcome.^7^ Many studies have observed that initial stroke severity and lesion volume fail to explain most of the variability in cognitive outcome.^7^ A possible reason is that, although AIS induces focal structural lesions, widespread alterations in functional connectivity networks occur and may further influence cognitive outcome and recovery. Consequently, alterations in large-scale functional brain networks rather than in single brain regions are thought to determine post-stroke cognitive outcome.^8,9^

The thalamus presents complex cortical and subcortical connections and is described to be a critical integrative node in large-scale functional brain networks.^10–14^ The thalamo-cortical loop develops before birth and plays a crucial role in the regulation of the development of cortical and thalamic territories.^15,16^ Different nuclei of the thalamus have been associated with specific cognitive functions, such as processing speed, attention, and executive functions.^12,14,17–19^ Hence, the thalamus and especially its connections are thought to play a crucial role in cognitive recovery after stroke.^20^

Resting-state functional magnetic resonance imaging (rs-fMRI) can be used to characterize large-scale systems such as the thalamo-cortical networks^19,21–24^ and measures the temporal correlation of blood-oxygen-level-dependent (BOLD) signals between different regions in the resting brain.^25^ This method has gained wide appeal owing to its simple application and reliability within and between individuals.^26,27^ Various resting-state networks can be drawn from a single scan while the child is at rest. This task-free procedure is particularly valuable in paediatric samples. Further, the investigation of resting-state networks has been shown to be of great clinical value, providing sensitive markers of disease.^28^

Studies in adult patients after stroke tend to agree that the strength of resting-state functional connectivity is associated with cognitive functions, including language, memory, executive functions, and attention.^29–33^ This is supported by findings of longitudinal studies revealing increases of functional connectivity over time which are associated with recovery of motor function.^34–36^ Only a few studies have investigated resting-state networks after AIS in a paediatric population.^8,9,37–39^ Our recent study^37^ and findings from another study^9^ have shown that the connectivity within the default mode network (DMN) and executive network is reduced in paediatric patients after AIS compared with controls, which in turn was correlated with reduced cognitive performance. Further, patients with hemiparesis after paediatric AIS showed reduced interhemispheric connectivity strength in the motor network compared to patients without hemiparesis and healthy controls.^40^ In studies that compared healthy controls to children with perinatal AIS, both weaker and stronger functional connectivity of the primary motor cortex to other brain regions was found.^38^

Functional connectivity measures between cortical and subcortical structures also predicted motor outcome after perinatal stroke.^41^ Altered thalamic volume, particularly in the non-lesioned hemisphere, has been associated with motor function,^42^ suggesting an important role for the thalamus in the developmental plasticity that determines motor function after perinatal stroke.

In a previous study, we demonstrated that the thalamus has functional connections to multiple cortical resting-state networks in healthy children, adolescents, and young adults and that stronger thalamo-cortical connectivity was associated with better cognitive performance in the healthy development between 5 and 25 years of age.^19^ However, the association between resting-state thalamo-cortical connectivity and cognition has never been investigated in patients after paediatric AIS. To this end, we aimed to investigate functional connectivity of the thalamo-cortical system and cognitive performance (including selective attention and executive functions) in patients after unilateral paediatric AIS and in healthy controls. Based on the literature presented previously, we hypothesized that (i) thalamo-cortical connectivity is reduced in patients after stroke compared with controls and that (ii) stronger thalamo-cortical connectivity is related to better cognitive performance (selective attention and executive functions).

## Materials and methods

### Participants

Participants were recruited as part of the HERO study^43^ examining functional reorganization after childhood stroke in a cross-sectional study design. The HERO study has been reviewed and approved by the Local Ethics Committee Bern (212/13), Switzerland.

Patients were identified by the Swiss Neuropaediatric Stroke Registry (SNPSR)—a multicentre, prospective, and population-based registry that includes children diagnosed with unilateral AIS under the age of 16 years.^44^ Patients met the following inclusion criteria: diagnosis of AIS (confirmed by MRI or CT) before the age of 16 years and at least 2 years prior to recruitment and older than 5 years of age at time of assessment to enable adequate compliance. The gap of 2 years between the AIS diagnosis and study examination was chosen to ensure that the patients were in the chronic stage, as the critical time window for recovery can extend beyond 1 year post-stroke.^45^ Exclusion criteria were active epilepsy (defined as seizures or treatment with anti-epileptic drugs during the 12 months prior to study participation), iron implants, claustrophobia, and behavioural problems that make an MRI scan impossible.

The control group, a sample of typically developing peers comparable in age and sex to the patient group, was recruited through advertisements on the hospital intranet and flyers. All healthy participants fulfilled the following inclusion criteria: (i) absence of neurological disease or psychiatric disorders, (ii) intelligence above IQ >85, and (iii) no contraindications for MRI (metal braces or metallic implants). All children and adolescents or their legal representative signed a written consent to participate in the study. Consent was obtained according to the Declaration of Helsinki. All examinations were performed at the University Hospital Bern, Inselspital, Switzerland. For more detailed information on the study design, see the previously published study protocol.^43^

Of the 29 patients recruited in the HERO study, 20 were included in the present analysis. Nine patients had to be excluded due to bilateral lesions (*n* = 4), retainer artefacts (*n* = 1), error in T_1_-weighted anatomical image or BOLD sequences (*n* = 2), or developmental delay or behavioural problems impeding compliance during the assessments (*n* = 2). Detailed clinical characteristics of the study participants are provided in Supplementary Table 1.

### Cognitive assessment

Cognitive functions were assessed with standardized neuropsychological tests conducted by trained psychologists or by study assistants and postgraduates (under supervision). We only included tasks with no fine motor components, as some patients had residual symptoms of hemiparesis. This ensured that reduced motor abilities did not influence cognitive performance. Neuropsychological assessments was performed at the Division of Neuropaediatrics, Development and Rehabilitation at the University Hospital, Inselspital, Bern, Switzerland, and were conducted within the same week as the MRI appointment at the Institute of Diagnostic and Interventional Neuroradiology, Inselspital, Bern, Switzerland.

To determine cognitive outcome, selective attention and executive functions were assessed: (i) selective attention was assessed with the cancellation subtest of the the Wechsler Intelligence Scale for Children and the Wechsler Adult Intelligence Scale, which is a visual-spatial search task. The three core dimensions of executive functions were also assessed:^46^ (ii) Working memory [spatial positioning subtest of the Learning and Memory Test (basic-MLT);^47^ (iii) inhibition and (iv) cognitive flexibility (colour–word inference test of the Delis Kaplan Executive Function System^48^). This test was conducted only with participants older than 8 years. This task includes four conditions where participants name coloured squares (condition 1), read words indicating colours printed in black ink (condition 2), name the incongruent ink colour of printed colour words (condition 3; inhibition) and switch between naming the ink colour and reading the colour words (condition 4; cognitive flexibility). This study contains the results from condition 3 (inhibition) which requires participants to inhibit a learned response (i.e. naming the colour of the words while ignoring their semantic content). Furthermore, this study contains the results of condition 4 (cognitive flexibility), where participants have to inhibit an overlearned response (i.e. naming colour of the words while ignoring their semantic content) but on top requires participants to demonstrate flexibility by set shifting. For more detailed information about neuropsychological tests see the previously published study protocol.^43^ Raw scores were used for analyses of functional connectivity measures, so that age could be fitted in the model. Each raw score was converted into a *Z*-score. To test whether cognitive performance differed between groups, scaled scores were analyzed with paired *t*-tests.

### MRI image acquisition and preprocessing

All participants were told to stay awake with their eyes closed and to remain as motionless as possible during the MRI scan. A head support system consisting of two pillows positioned on both sides of the head was used to minimize head motion. Earplugs were given to the participants to minimize the scanner noise.

### Structural images

MRI data were acquired on a 3 T Magnetom Verio scanner (Siemens, Erlangen, Germany). The structural images were aquired using a three-dimensional magnetization-prepared rapid gradient-echo T_1_-weighted sequence [repetition time (TR) = 2530 ms, echo time (TE) = 2.96 ms, inversion time = 1100 ms, and a flip angle (FA) = 7°, field of view (FOV) 256×256 mm^2^, matrix dimension 256×256, leading to an isovoxel resolution of 1×1×1 mm^3^, acquisition time (TA) = 5.05 min).

Lesion-related characteristics were determined by a board-certified neuroradiologist (N.S.). Lesion laterality was classified depending on the affected hemisphere (left, right or bilateral) and lesion location was divided into three categories (cortical, subcortical or combined cortical and subcortical, according to this previous work^49^). Ischaemic lesions were manually segmented (slice by slice) on the structural images (T1) to calculate the volume of the affected brain tissue using the open-source software 3D Slicer, version 4 (https://www.slicer.org). Affected brain tissue was selected based on hypointensity on the T_1_-weighted images. Both CSF filled areas as well as encephalomalacic areas and gliotic parts may appear hypo-intense on T_1_-weighted as well. Only post-ischaemic defects were evaluated and the adjacent CSF spaces were not included in the analysis. Lesion size was defined as the affected brain tissue in relation to the total brain volume. Total brain volume was calculated using the MATLAB-based toolbox Statistical Parametric Mapping (SPM12). More in detail, the structural images were then transformed into the standard Montreal Neurological Institute (MNI) template and then segmented into grey matter, white matter, and CSF. In the processing steps for segmentation within SPM12, there are efforts to overcome problems of gliotic parts (e.g. as may be originated by lacune) that eventually would be associated to CSF tissue class. Notably, SPM12 is widely used for segmentation of anatomical images.^50,51^ Furthermore, the segmented lesions were integrated as masks in the preprocessing pipeline. Resting-state connectivity was only measured in healthy tissue.

### Functional images

Functional images were acquired using a T_2_*-weighted multi-band, simultaneous excitation echo planar imaging (mb-EPI) sequence (TR = 300 ms, TE = 30 ms, FA = 30°, FOV 230×230 mm^2^, matrix dimension 64×64, 32 axial slices positioned along the anterior commissure and the posterior commissure with a slice thickness of 3.6 mm, gap 0 mm, leading to an isovoxel resolution of 3.6×3.6×3.6 mm^3^). The TA = 5.06 min, and each scan consisted of 1000 image volumes.

### Preprocessing

Preprocessing of fMRI data was performed using FMRIB Software Library (FSL; http://www.fmrib.ox.ac.uk/fsl^52–54^). The fMRI time series for each subject was pre-processed as follows (pipeline recommended elsewhere^55,56^): (i) rigid body realignment; (ii) within-subject intensity normalization (in which the intensity across all voxels was scaled to a norm value of 1000. The BOLD signal is then represented in a mode 1000 scale (10 units = 1% BOLD); (iii) coregistration of functional images to each of the individual anatomical images. All functional images were resampled to an isovoxel space with 3 mm × 3 mm × 3 mm spatial resolution; (iv) finally, functional images were transformed to the standard MNI template.

### Identification and treatment of motion artefacts by censoring functional MRI time series

Head motion could compromise the computation of correlations in resting-state functional connectivity fMRI. Steps to identify and eventually remove motion artefacts during fMRI acquisition were therefore taken.^55,56^ Framewise displacement (FD) was used to record the absolute amount of motion during scanning and the relative measure expressed as root mean square value of the differentiated BOLD time series (by backward differences, DVARS). This DVARS measure captures the change in signal intensity from one volume to the adjacent previous volume.

With these two measures, each individual fMRI time series was censored (i.e. scrubbing). The following criteria were used to identify and quantify ‘artefact-affected volumes’: FD > 0.2 and DVARS > 0.38, i.e. a ‘geometrical’ and a ‘physiological’ surrogate for the motion-related signal component. The detailed procedure of censoring volumes and its effect on the quality of the data of each participant are defined in more detail in the Supplementary Materials (Supplementary Fig. 1).

### Nuisance regressors

A set of six motion estimates [R = (x, y, z, pitch, yaw, roll)] were stored for each subject during the realignment step. These motion estimates were the basis for the computation of three additional motion-related indices: their squares (R2), (Rt—1) and (R2t—1), where t and t—1 apply to the current and immediately preceding time point of the fMRI time series.

In summary, this procedure results in 24 motion-related indices that were further used as nuisance regressors in the multiple regression analyses. Tissue-based signals from average signal across voxels within a ventricular mask for the CSF signal and white matter mask for the white matter signal were also used as nuisance regressors. Finally, global signal regression^57,58^ was included as an effective procedure to remove motion-related artefacts.^55^ A set of 27 nuisance regressors were included in the multiple regressions.

#### Processing of neuroimaging data and statistical analyses

To define a mask for the thalamus (region 10 and 49; left and right thalamus) the Harvard–Oxford cortical and subcortical structural atlases were adopted (https://fsl.fmrib.ox.ac.uk/fsl/fslwiki/Atlases). In total, 672 voxels comprised in the final thalamus mask, which was down-sampled into matching 3×3×3 mm^3^ in MNI space.

#### Identifying thalamic connectivity patterns

To identify thalamic connectivity patterns, the same strategy as in previous work was adopted.^19,22^ First, functional images from patients with lesions in the right hemisphere (*n* = 4) were flipped along the midsagittal plane, so that the affected hemisphere corresponded to the left hemisphere in the whole patient sample. Then, time series from all voxels of the thalamus were correlated with all voxels of the rest of the brain using Pearson’s correlation coefficient. Six hundred and seventy-two seed-based correlation maps (connectivity maps) for each participant resulted from this procedure. All correlation maps were then Fisher-transformed to *Z*-scores [*Z* = 0.5 * ln (1 + r/1−r)]. And concatenated into a single 4D data set with the dimensions of 61×73×61×26 880 voxels (a single volume has 61×73×61 voxels; 672 volumes for each of the 40 participants). Then, the independent component analyses (ICAs) were calculated on the resulting seed-based correlation maps using FSL’s Multivariate Exploratory Linear Optimized Decomposition into Independent Components to identify spatially distinct connectivity maps. The resulting 4D data set was decomposed into 20 spatially independent components (ICs).^22^ From this set of ICs, we were able to identify nine well-known brain networks for further analysis.

### Thalamo-cortical connectivity and statistical analysis

To quantify the functional connectivity between each thalamic voxel and brain network, we first determined the contributions of each IC network with the individual thalamic connectivity maps (subject level). These individual thalamic connectivity maps and the IC network maps were then transformed into vectors, and linear regression was used to quantify functional connectivity for each voxel across all individuals. In particular, linear regression was used to measure the contribution from each IC to the voxel’s seed-based correlation map (for each given voxel in the thalamus). The regression analysis was performed for all of the 672 voxels in the thalamus, and nine beta values were obtained for each voxel. A voxel-wise, one-sample *t*-test was performed on every network’s thalamic beta map across subjects to find all significant clusters in the thalamus for each network separately.

Potential differences between the groups for the ICA components produced were assessed using paired *t*-tests. To examine the impact of cognitive functions (processing speed, selective attention, inhibition, working memory, cognitive flexibility) on thalamo-cortical connectivity strength in patients, we applied multivariate linear regressions. Cognitive variables were integrated as independent variables and connectivity strength values as dependent variables, with age and lesion size as covariates.

We applied false discovery rate (FDR)-adjusted *P*-values to correct for multiple testing. An FDR-adjusted *P* < 0.05 was considered statistically significant. All analysis steps are similar to the procedure used previously in an adult population^22^ and in a paediatric population.^19^

The significant clusters in the thalamus were identified and labelled according to the thalamus atlas of Morel and Krauth.^59,60^ This atlas is built in MNI space and includes 40 small thalamic nuclei. Analyses were performed with MATLAB 9.2 (MathWorks, Sherborn, MA, USA). We focused mainly on the largest of the 40 nuclei present in the Morel atlas without differentiating finer subdivisions of the different nuclei to minimize identification errors.

### Data availability

Individual patient data, including neuroimaging data, cognitive data, and some demographical variables are available upon reasonable request after signing a confidentiality statement and a data sharing agreement and in accordance with data privacy statements signed by all patients.

## Results

### Descriptive and cognitive measures

Details of the clinical characteristics of the study participants are provided in Supplementary Table 1. Patients and healthy controls did not differ in terms of age and sex, as groups were matched for these variables (Table 1). Patients after paediatric stroke had significantly lower scores for selective attention [t(38) = 2.05, *P* = 0.048], working memory [t(38) = 2.51, *P* = 0.016], and inhibition [t(38) = 2.18, *P* = 0.036] than controls. There was no significant between-group difference in cognitive flexibility (Table 1). However, there is a trend, with a moderate effect size, for cognitive flexibility to be lower in children after stroke.

**Table 1:** Demographic and cognitive variables of patients and healthy controls

	Patients	Controls	*t(df)*	*p*	Cohen’s *d*
	Mean (SD)	Mean (SD)			
Sex, *n* (%)					
Female	8 (40.0)	8 (40.0)			
Male	12 (60.0)	12 (60.0)			
Age at assessment (years)	15.01 (4.28)	15.2 (4.10)	0.14 (39)	0.900	
Selective attention (SS)	8.09 (3.30)	11.58 (2.58)	2.05 (39)	0.048*	0.64
Range	2–13	6–16			
Working memory (PR)	43.37 (31.84)	69.68 (29.21)	2.51 (37)	0.016*	0.79
Range	3–100	8–99			
Inhibition (SS)	9.32 (3.11)	11.21 (2.18)	2.18 (37)	0.036*	0.71
Range	2–13	6–14			
Cognitive flexibility (SS)	9.63 (3.58)	11.58 (2.29)	2.00 (37)	0.052	0.65
Range	3–13	8–15			

SD, standard deviation; SS, scaled score; PR, percent range, * *p* < 0.05

### Thalamo-cortical networks

**Figure 1:**
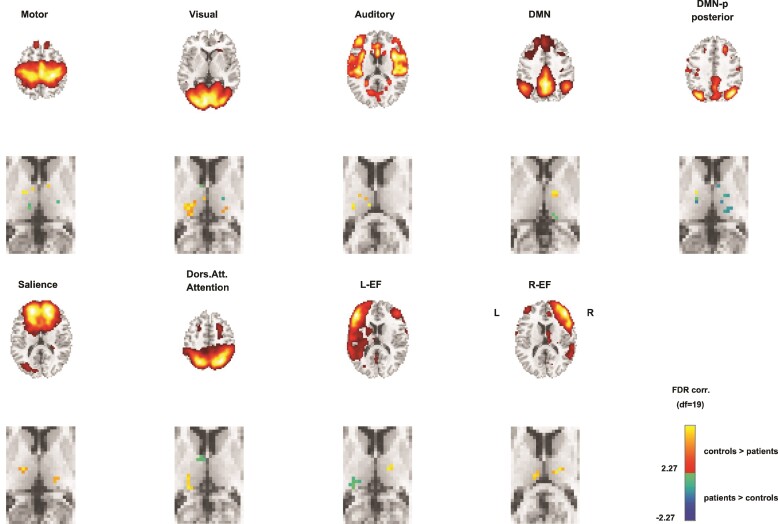
**Group differences in thalamo-cortical networks.** Nine cortical networks from group-level ICAs (first and third row) and thalamic clusters that differed between groups for each resting-sate network (second and fourth row) are depicted. (i) Motor network; patients showed lower thalamo-cortical connectivity in the mediodorsal nucleus and nuclei from the anterior group and higher thalamo-cortical connectivity in nuclei from the lateral group. (ii) Visual network; patients showed lower thalamo-cortical connectivity in the pulvinar, ventral lateral, ventral posterior, mediodorsal and lateral posterior nuclei. (iii) Auditory network; patients showed lower thalamo-cortical connectivity in the lateral and medial nuclei, including the lateral dorsal and mediodorsal nuclei. (iv) DMN; patients showed lower thalamo-cortical connectivity in the pulvinar and higher thalamo-cortical connectivity in the mediodorsal nucleus. (v) Posterior DMN; patients showed lower thalamo-cortical connectivity in the lateral nuclei and in the pulvinar. (vi) Salience network; patients showed lower thalamo-cortical connectivity in the mediodorsal nucleus and the pulvinar. (vii) Dorsal attention network; patients showed lower thalamo-cortical connectivity in the pulvinar, and mediodorsal nucleus and higher thalamo-cortical connectivity in the anteroventral nucleus. (viii) Right and left executive network; patients showed lower thalamo-cortical connectivity in the mediodorsal nucleus, pulvinar and in the lateral group. (ix) Left executive network: patients showed higher thalamo-cortical connectivity in the pulvinar. *Notes*. DMN, default mode network; L-EF, left executive network; R-EF, right executive network. The right side of the brain is on the right side of the image. All sub-regions of the thalamus are thresholded at FDR-corrected *P* = 0.05. Only FDR-corrected voxels are depicted in the Figure.

Analyses revealed nine networks involved in cognitive, sensory-, and motor-related processing. These networks were comparable with the networks that have previously been observed in rs-fMRI studies in adults^22^ and children.^19,22,26,27,61^ These nine networks represent the DMN, the posterior DMN, left and right executive, auditory, dorsal attention, motor, salience, and lateral visual networks (Fig. 1).

### Group differences in thalamo-cortical networks

Figure 1 summarizes group differences in thalamo-cortical connectivity. Analyses revealed significant differences between patients and controls in thalamo-cortical connectivity in all resting-state networks, including the motor, auditory, visual, DMN, posterior DMN, salience, left/right executive and dorsal attention networks.

For the motor network, patients showed lower thalamo-cortical connectivity in the mediodorsal nucleus and in nuclei from the anterior group, whereas higher thalamo-cortical connectivity was found in nuclei from the lateral group. For the visual network, patients showed lower thalamo-cortical connectivity in the pulvinar, ventral lateral, ventral posterior, mediodorsal,and lateral posterior nuclei. For the auditory network, patients showed lower thalamo-cortical connectivity in the lateral and medial nuclei, including the lateral dorsal and mediodorsal nuclei. For the DMN, patients showed lower thalamo-cortical connectivity in the pulvinar, whereas higher thalamo-cortical connectivity was found in the mediodorsal nucleus. For the posterior DMN, patients showed lower thalamo-cortical connectivity in the lateral nuclei and the pulvinar. For the salience network, patients showed lower thalamo-cortical connectivity in the mediodorsal nucleus and the pulvinar. For the dorsal attention network, patients showed lower thalamo-cortical connectivity in the pulvinar and mediodorsal nucleus, whereas higher thalamo-cortical connectivity was found in the anteroventral nucleus. For the right and left executive network, patients showed lower thalamo-cortical connectivity in the mediodorsal nucleus, pulvinar, and in the lateral group. For the left executive network, patients showed in addition higher thalamo-cortical connectivity in the pulvinar.

To further ensure that the observed group differences in thalamo-cortical connectivity strength were not originated by the implemented lesion masks, a further analysis was conducted. We applied the same lesion masks as for the AIS population to the images of the healthy controls. The ‘lesion masks’ for the healthy controls were exactly the same with respect to the total number of voxels and were exactly positioned at the same location as for the patients. A paired *t*-test was conducted to compare the thalamo-cortical connectivity between healthy controls and healthy controls with the implemented lesion masks. Analyses revealed no substantial differences in the thalamo-cortical connectivity strength (supplementary Fig. 2).

Lastly, as there were three patients in the sample with thalamic infarctions, we conducted a supplementary analysis investigating the thalamo-cortical networks without these three patients (*N* = 17 i.e. excluding the patients who have lesions in the thalamus). These results are shown in supplementary Fig. 2.

### Thalamo-cortical connectivity and cognition

**Figure 2:**
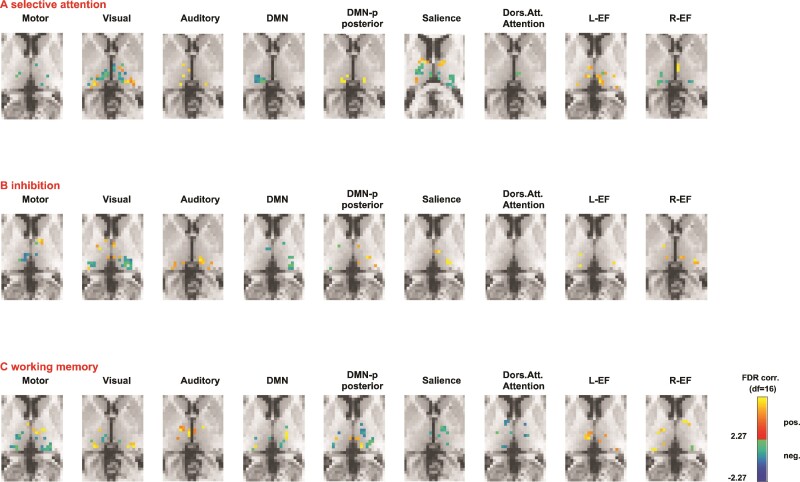
**Cognition and thalamo-cortical networks.** Association between thalamo-cortical connectivity and cognitive performance in paediatric patients after stroke. (A) Selective attention showed significant associations with connectivity for all resting networks; positive associations were found in the intralaminar, lateral dorsal, lateral posterior, and the mediodorsal nucleus; negative associations were found in the pulvinar and the anteroventral nucleus. (B) Inhibition showed significant associations with connectivity for all resting networks; positive associations were found in the mediodorsal, intralaminar, ventral lateral nucleus, and pulvinar; negative associations were found in the anteroventral nucleus, mediodorsal nucleus, and sub-nuclei of the lateral group. (C) Working memory showed significant associations with connectivity for all resting-state networks; positive associations were found in the mediodorsal, intralaminar, ventral lateral, and pulvinar; negative associations were found in the mediodorsal nucleus, intralaminar, and ventral lateral nucleus. *Notes*. WM, working memory; DMN, default mode network; L-EF, left executive network; R-EF, right executive network. The right side of the brain is on the right side of the image (including the data that was flipped). Illustrated significant clusters in different sub-nuclei of the thalamus result from the multivariate linear regression with lesion size and age as covariates. All sub-regions of the thalamus are thresholded at FDR-corrected *P* = 0.05. Only FDR-corrected voxels are depicted in the Figure.

Figure 2 summarizes the association between thalamo-cortical connectivity and cognitive performance in patients. Multivariate linear regression (lesion size and age as covariates) revealed significant associations between cognitive performance and the strength of thalamo-cortical connectivity in the motor, auditory, visual, DMN, posterior DMN, salience, left/right executive, and dorsal attention network after paediatric stroke (Fig. 2; supplementary Table 2).

Multivariate linear regressions in the patient sample with selective attention as independent variable and thalamo-cortical connectivity as dependent variable (lesion size and age as covariates) revealed significant associations for all resting networks. Positive associations were found in the intralaminar, lateral dorsal, lateral posterior, and the mediodorsal nucleus; negative associations were found in the pulvinar and the anteroventral nucleus (Fig. 2; supplementary Table 2). Multivariate linear regressions with inhibition as independent variable and thalamo-cortical connectivity as dependent variable (with lesion size and age as covariates) revealed significant associations for all resting networks. Specifically, positive associations were found in the mediodorsal, intralaminar, ventral lateral nucleus, and pulvinar. Negative associations were found in the anteroventral nucleus, mediodorsal nucleus, and sub-nuclei of the lateral group (Fig. 2; supplementary Table 2). Multivariate linear regressions with working memory as independent variable and thalamo-cortical connectivity as a dependent variable (lesion size and age as covariates), revealed significant associations for all resting networks. Specifically, positive associations were found in the mediodorsal, intralaminar, ventral lateral, and pulvinar. Negative associations occurred in the mediodorsal nucleus, intralaminar, and ventral lateral nucleus (Fig. 2; supplementary Table 2). Cognitive flexibility showed no significant associations with thalamo-cortical connectivity. All reported results remained significant after FDR correction. Voxels and *t* values are listed in Table S2.

## Discussion

The present cross-sectional study aimed to compare resting-state thalamo-cortical connectivity between patients after unilateral paediatric AIS and healthy controls. We further explored the relationship between thalamo-cortical connectivity and cognitive outcome in patients after paediatric stroke. Cognitive functions were significantly reduced in patients compared to healthy controls, supporting the findings of previous studies.^62–64^ According to our primary hypothesis, we found significantly weaker thalamo-cortical connectivity for the motor, auditory, visual, DMN, salience, left/right executive, and dorsal attention network in patients compared with controls. In additionally, we also found stronger thalamo-cortical connectivity in the DMN, motor, dorsal attention, and the left executive network in patients compared with controls. Stronger thalamo-cortical connectivity for patients compared with controls was however found in different thalamic nuclei, pointing to hypo- as well as hyperconnectivity in the patients sample in different parts of the thalamus. According to our secondary hypothesis, thalamo-cortical connectivity strength was positively associated with selective attention, inhibition and working memory. In accordance with the results of the group analyses, we also found negative associations between cognitive functions and thalamo-cortical connectivity strength. The significant relationship between thalamo-cortical connectivity and cognitive functions after paediatric stroke has never been described previously.

### Group differences in thalamo-cortical networks

Nine resting-state networks were identified and were comparable to the networks that have previously been observed in rs-fMRI studies.^19,22,26,27,61^ Significant differences between patients and controls were found for all thalamo-cortical networks within all three major nuclear regions, as well as the pulvinar and the intralaminar nuclei. Furthermore, between-group differences were observed in the ipsilesional, as well as in the contralesional hemisphere, which supports the assumption that focal structural brain damage can lead to alterations in remote regions.^33,41,65^

Specifically, patients showed weaker thalamo-cortical connectivity in the motor, auditory, visual, DMN, salience, left/right executive, and dorsal attention network. Our finding of reduced functional connectivity strength in patients is in line with previous findings in paediatric^37,38^ and adult stroke populations.^23,36,66–69^ Reduced connectivity strength might be interpreted as sustained interruption of functional network efficiency. Interestingly, we also found stronger thalamo-cortical connectivity in other thalamic subdivisions in the DMN (medial nuclei), motor (lateral nuclei), dorsal attention (anterior nuclei), and the left executive network (posterior nuclei) in patients compared with controls. Stronger thalamo-cortical connectivity was however found in different thalamic nuclei. The finding of higher thalamo-cortical connectivity strength in patients than in controls may reflect a compensatory effect due to reorganizational processes occurring after brain damage.^9^ Thus, alterations in thalamo-cortical connectivity strength after paediatric stroke seem to be in both directions, with stronger as well as weaker thalamo-cortical connectivity, likely representing different patterns of functional connectivity after paediatric stroke. Our findings of increased and decreased functional thalamo-cortical connectivity strength thereby build on previous literature in stroke populations reporting increased and decreased resting-state functional connectivity on a cortical level.^9,33,38,70^

In addition, it has been shown previously that both cortical and subcortical areas, such as the thalamus, show alterations in connectivity strength after stroke affecting both the ipsi- and the contralesional hemisphere.^33,41,71^ One possible explanation derives from work describing the process of diaschisis,^72,73^ which showed that stroke can also induce changes in the function and physiology of brain regions distant from the site of anatomical damage. This concept is supported by studies on homogeneous patient groups (e.g. ischaemic strokes in basal ganglia or periventricular venous infarction), which demonstrated altered connectivity strength in structures remote from the lesion.^41,65^ Alterations of functional connectivity in the contralesional hemisphere seen in our study might therefore support theories of network organization of the brain.^71,74^

The fact that our patients were assessed in the chronic post-stroke phase (at least 2 years after AIS) highlights that there are persistent alterations in functional thalamo-cortical connectivity even years after the stroke. A growing number of studies indicate that stroke induces changes of functional connectivity within and between resting-state networks in the chronic phase^29,65,75^ and that these changes correlate with chronic impairment post-stroke.^65,76^ The question whether the motor networks is affected by a hemiparesis, was addressed in one of our previous studies.^40^ We investigated functional connectivity in paediatric patients after stroke with hemiparesis compared with patients with a good clinical outcome and healthy controls. Patients with hemiparesis showed lower interhemispheric connectivity strength in the motor network compared to patients with good clinical outcome and controls.^40^

To further ensure that the observed group differences in thalamo-cortical connectivity strength were not originated by the implemented lesion masks, a further analysis with the same lesion masks was conducted. Analyses investigating the effect of the implemented lesion masks revealed no substantial differences in the thalamo-cortical connectivity strength (Supplementary Fig. 2). If the implementation of the lesion mask were influencing the strength of the thalamo-cortical connectivity, then differences in the thalamo-cortical connectivity should have been observed. Finally, as there were three patients in the sample with thalamic infarctions, we conducted a supplementary analysis investigating the thalamo-cortical networks without these three patients. Analyses revealed that there were only minimal changes in the *t*-test comparing patients and controls. Thus, these three patients did not substantially affect the results presented in the study.

### Thalamo-cortical connectivity and cognition

Selective attention, inhibition and working memory was positively associated with functional thalamo-cortical connectivity strength in networks that have previously been related to cognitive processing (posterior DMN, salience, dorsal attention, and executive network).^77^ Further, our analyses also revealed significant positive associations between thalamo-cortical connectivity and cognitive functions in networks involved in visual, auditory, and motor-related processes in our patient sample. This might be because most cognitive functions arise not solely from a particular brain area but from networks spanning multiple distributed regions.^78,79^ Furthermore, the cognitive tasks require not only specific cognitive skills but also sensory-related functions such as visual and auditory related processing.^80,81^ Multiple processes underlie the performance of a cognitive task and no task is a pure measure of a single cognitive process.^80,81^ We also found negative associations between cognitive functions and thalamo-cortical connectivity for several networks. These associations were, however, in different parts of the thalamus, pointing towards a specialization of certain thalamic sub-regions.

The cancellation task used to measure selective attention is a visuo-spatial search task. Its performance was associated with multiple thalamo-cortical networks, with a predominant pattern of significant correlations for the visual, salience, and executive networks. For the visual network, positive associations with selective attention were found in the pulvinar, meaning that the better the selective attention, the higher the connectivity strength in this thalamic nucleus. The pulvinar has widespread reciprocal connectivity to different cortical areas, such as occipital, temporal, parietal and frontal regions. The pulvinar has previously been related to visuo-spatial attention processing.^12,82^ For the salience and executive network, positive associations with selective attention were found in the anterior nuclei and the mediodorsal nucleus of the thalamus, which are known to influence cognitive processes, including attention and executive functions.^11,83,84^ In addition, significant positive associations were observed with the auditory and motor-related network, possibly indicating that sensory-related thalamo-cortical networks may also be relevant for selective attention.

Inhibition is a core dimension of executive functions^46^ and was measured with colour–word interference tasks (comparable with a Stroop task). Performance of this task was associated with multiple thalamo-cortical networks, with a predominant pattern of positive correlations for the salience, dorsal attention and left and right executive networks (Fig. 2, supplementary Table 2). Specifically, the analyses revealed that the better the inhibition, the stronger the thalamo-cortical connectivity in these networks. Significant positive associations with inhibition were also observed for the visual, auditory, and motor-related networks. This might indicate that sensory-related thalamo-cortical networks could also be relevant for inhibition. In line with this proposition, inhibitory performance has been attributed to spatially distributed functional networks.^79^ Higher-order cognitive capacities have been shown to depend on complex large-scale brain systems.^85^

Working memory is another core dimension of executive function^46^ and was measured with a visuo-spatial location task. Working memory performance was associated with multiple thalamo-cortical networks, with a predominant pattern of positive correlations for the left and right executive networks (Fig. 2, supplementary Table 2). Visuo-spatial working memory function is based on a complex network of frontal, parietal, occipital and temporal areas.^86^ Significant positive associations were also observed for the visual, auditory and motor-related networks, indicating that sensory-related thalamo-cortical networks also seem to be relevant for visuo-spatial working memory.

Finally, the question arises why cognitive flexibility was not significantly associated with thalamo-cortical connectivity. First, from a statistical point of view, this correlation did not survive FDR correction but showed, however, non-corrected significant correlations. Different assumptions might be possible as to why there were only weak correlations between thalamo-cortical connectivity and cognitive flexibility. First, as there was only a trend for cognitive flexibility to be lower in patients after stroke, this cognitive domain seems to be less affected compared the other cognitive domains. Accordingly, studies with different clinical samples have shown that cognitive flexibility (assessed with the colour–word inference test) was less affected than inhibition.^87–89^ Since cognitive flexibility is the fourth and last condition of the colour–word inference test, patients might benefit from some familiarization or even learning effect occurring throughout the two conditions, leading to better cognitive flexibility than inhibition.^90^

Our findings therefore support the assumption, that higher-order cognitive functions after childhood AIS depend on the integrated processing of multiple large-scale functional brain networks,^85^ with the thalamus functioning as an integrative hub for cortical networks.^10^ Post-stroke cognitive outcome in our sample may reflect altered functional connections across multiple networks.

### Limitations

The findings of the present study should be viewed in the light of some limitations. First, our sample size was relatively small. Therefore, further research is needed to replicate our findings in a larger cohort and confirm that brain connectivity in the thalamo-cortical network is related to long-term cognitive outcome. Second, it is difficult to precisely locate each thalamic nucleus using a standardized template since thalamic nuclei differ in size and locations and there is rather low spatial resolution of the functional images. Further, the thalamus atlas was constructed from histological data on adults^60^ and afterwards reconstructed in the MNI space.^59^ Therefore, a potential mismatch is expected. We therefore focused mainly on the largest of the 40 nuclei present in the Morel atlas, without differentiating finer subdivisions of the different nuclei. Moreover, in patients with large lesions (e.g. involving cortical and subcortical brain regions), it may be in general challenging to define the thalamus mask due to midline shift. However, we did not face difficulties in defining the thalamus mask for these patients during coregistration. We conducted a supplementary analysis investigating the thalamo-cortical networks excluding the patients who had lesions in the thalamus (*N* = 17). This analysis revealed no substantial differences in the thalamo-cortical connectivity strength (Supplementary Fig. 3). Finally, cross-sectional study designs provide information at a given time point in patients after stroke. However, as functional networks and cognitive outcomes likely change over time during development, longitudinal study designs are needed to gain a deeper insight into the development of thalamo-cortical networks and their possible relation to cognitive rehabilitation in children and adolescents.

## Conclusion

To the best of our knowledge, this is the first set of results showing a relationship between thalamo-cortical functional connectivity and cognitive functions (selective attention, working memory, and inhibition) after childhood stroke. Our data provide evidence that the interaction between different sub-nuclei of the thalamus and multiple cortical networks is crucial for post-stroke cognitive functions. Furthermore, the data support the idea that large-scale networks such as the thalamo-cortical system may help to explain the variability in cognitive outcomes after childhood AIS. Future investigations of recovery mechanisms after brain injury and the development of novel interventions and therapies should consider the network perspective of brain functions.

## Supplementary Material

fcac110_Supplementary_Data

## Abbreviations

AIS =: arterial ischaemic stroke
BOLD =: blood-oxygen-level-dependent
DMN =: default mode network
FDR =: false discovery rate
FD =: framewise displacement
fMRI =: functional magnetic resonance imaging
IC =: independent component
ICAs =: independent component analyses
IQ =: intelligence quotient
MNI =: Montreal Neurological Institute
rs-fMRI =: resting-state functional MRI
SPM12 =: statistical parametric mapping
