# Supplementary material for: Cognitive outcome is related to functional thalamo-cortical connectivity after paediatric stroke
Source: Brain Commun. 2022 Apr 28;4(3):fcac110. doi: 10.1093/braincomms/fcac110 (PMC9122536; doi:10.1093/braincomms/fcac110)
Supplement: fcac110_Supplementary_Data [file fcac110_supplementary_data.pdf]

Supplementary material for

Cognitive outcome is related to functional thalamo-cortical connectivity after pediatric stroke

Leonie Steiner[1,2†], Andrea Federspiel[3†], Nedelina Slavova[4], Roland Wiest[4], Sebastian Grunt[1], Maja Steinlin[1], Regula Everts[1,5]

[†] These authors contributed equally to this work.

For Review Only

This file includes:

Supplementary Table 1 & 2

Supplementary Figure 1 – 3

Supplementary information for head motion and fMRI processing steps

Supplementary Table 1. Characteristics of individual participants

Number	Sex (m/f)	Age at examination (y)	Age at stroke (y)	Time since stroke (y)	Stroke side	Stroke location	Lesion size (mm³)	Hemiparesis yes/no
1	f	6.75	4.00	2.08	left	cortical	0.005	no
2	m	22.67	14.50	8.17	left	subcortical	3.791	no
3	f	20.83	10.41	10.42	left	subcortical (thalamus involved)	0.006	yes
4	f	15.50	11.75	3.58	right	subcortical (thalamus involved)	0.006	no
5	m	18.42	6.25	12.08	left	combined cortical/subcortical (thalamus involved)	1.079	yes
6	f	19.92	3.42	10.50	right	combined cortical/subcortical	0.346	yes
7	f	15.33	7.83	7.41	left	subcortical	0.023	no
8	m	11.42	3.50	7.83	left	subcortical	0.008	yes
9	m	13.17	5.75	7.33	right	combined cortical/subcortical	3.471	no
10	m	18.75	14.33	4.42	left	subcortical	0.050	no
11	m	8.75	5.33	3.42	left	combined cortical/subcortical	7.743	yes
12	m	9.42	1.50	7.92	left	subcortical	0.998	yes
13	m	11.67	6.67	4.92	right	combined cortical/subcortical	0.011	no
14	f	18.50	14.67	3.75	left	subcortical	0.004	no
15	f	20.75	9.83	10.92	left	combined cortical/subcortical	11.682	yes
16	m	9.50	0.00	9.50	left	cortical	0.018	no
17	f	13.42	0.00	13.42	left	cortical	2.646	yes
18	m	10.75	0.00	10.75	left	combined cortical/subcortical	0.017	no
19	m	16.75	1.17	15.50	left	subcortical	0.086	no
20	m	23.08	15.58	7.5	left	subcortical	1.039	yes

Note. Calculation of lesion size: volume of lesion/total intracranial volume × 1000. SC, subcortical; C, Cortical; L, left; R, right; y, years; m, male; f, female; The Pediatric Stroke Outcome Measure (PSOM) was used to define at the time of MRI scanning. We used the sensorimotor subscale to classify the presence of hemiparesis (0 = no sensorimotor deficit; 0.5 = mild deficit, with normal function; 1 = moderate deficit, with decreased function; 2 = severe deficit with no function). Patients with scores greater than 0.5 on the sensorimotor subscale were classified as having hemiparesis. Patients with a score of zero on all subscales were classified as having a good clinical outcome.

For Review Only

For Review Only

Identification and treatment of head motion

The identification and subsequent treatment of potential subjects' head motion during the acquisition of fMRI time series was addressed in the present study. We decided to follow the processing pipeline as described in more detail in Power JD, et al.[1, 2] that was adopted in a previous work[3]. In summary, fMRI time series of each subject was pre-processed as follows: 1) rigid body realignment; 2) within-subject intensity normalization. In this step the intensity across all voxels is scaled to a norm value of 1000. This implies that the BOLD signal afterwardsis represented in a mode 1000 scale (10 units = 1% BOLD); 3) coregistration of fMRI data to each individual anatomical MP-RAGE scans. During this step all fMRI data are resampled to a isovoxel space with 3 mm x 3 mm x 3mm spatial resolution; 4) the functional images were then transformed to the standard Montreal Neurological Institute (MNI) template.

Contrary to the pre-processing steps described in [1], we did not apply the slice-time correction to the raw fMRI time series. All fMRI data were acquired using a multi-band, simultaneous excitation EPI sequence with TR=300 ms (e.g. multiple slices are acquired at the same time); the effect of interpolation to temporally align each slice to the start of each volume is therefore negligible.

During the realignment step a set of 6 motion estimates (R = [x,y,z, pitch, yaw, roll]) are stored for each subject. These motion estimates are the basis of computation of additional motion-related indices such as: their squares (R^2), (R_{t-1}) and (R^2_{t-1}), where t and t − 1 refer to the current and immediately preceding timepoint of the fMRI time serie.

Altogether, these 24 motion-related indices are further used as nuisance regressors in the multiple regressions. Additionally, tissue-based signals from average signal across voxels within a ventricular mask for the CSF signal and white matter mask for the WM signal were also used as nuisance regressors.

Lastly, in the present study we included global signal regression (GSR) [4, 5] as an effective procedure to reduce motion-related artifacts [1]. In summary, a total set of 27 nuisance regressors were included in the multiple regressions.

For Review Only

Censoring fMRI time series

In order to identify and quantify volumes of the fMRI time series of each subject that potentially were originated by head motions, we calculated two scalar measures: 1) framewise displacement (FD) [6] and 2) the rate of change of BOLD signal across the entire brain at each volume and each volume of immediately preceding timepoint (DVARS) [6]. To calculate DVARS, the volumetric time series is differentiated (by backwards differences) and RMS signal change is calculated over the whole brain [7].

In the present analysis we used the following criteria to identify and quantify "artifact-affected volumes": FD>0.2 AND DVARS>38 i.e. a "geometrical" and a "physiological" surrogate of motion-related signal component. With these cuts we are censoring 0.12 % of the data of patients and 0.21 % of the data of controls (i.e. 1000 volumes with TR=300 ms) and we are still within the recommended range of adequate resting state scan length of ~5 min [8].

For Review Only

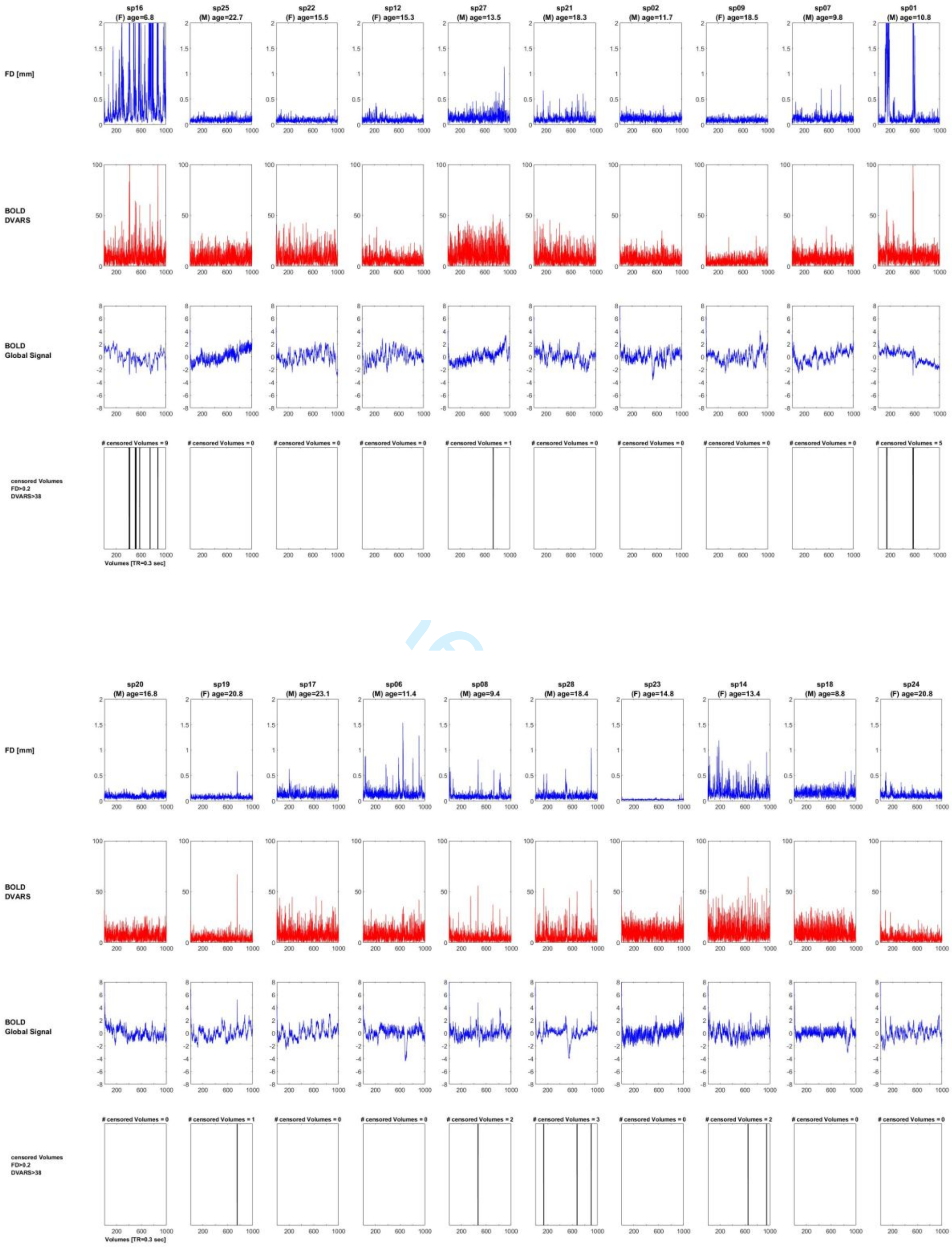
sp16 (F) age=6.8
sp25 (M) age=22.7
sp22 (F) age=15.5
sp12 (F) age=15.3
sp27 (M) age=13.5
sp21 (M) age=18.3
sp02 (M) age=11.7
sp09 (F) age=18.5
sp07 (M) age=9.8
sp01 (M) age=10.8
FD [mm]
BOLD DVARS
BOLD Global Signal
censored Volumes FD>0.2 DVARS>38
censored Volumes = 9
censored Volumes = 0
censored Volumes = 1
censored Volumes = 5
Volumes [TR=0.3 sec]
sp20 (M) age=16.8
sp19 (F) age=20.8
sp17 (M) age=23.1
sp06 (M) age=11.4
sp08 (M) age=9.4
sp28 (M) age=18.4
sp23 (F) age=14.8
sp14 (F) age=13.4
sp18 (M) age=8.8
sp24 (F) age=20.8
censored Volumes = 2
censored Volumes = 3

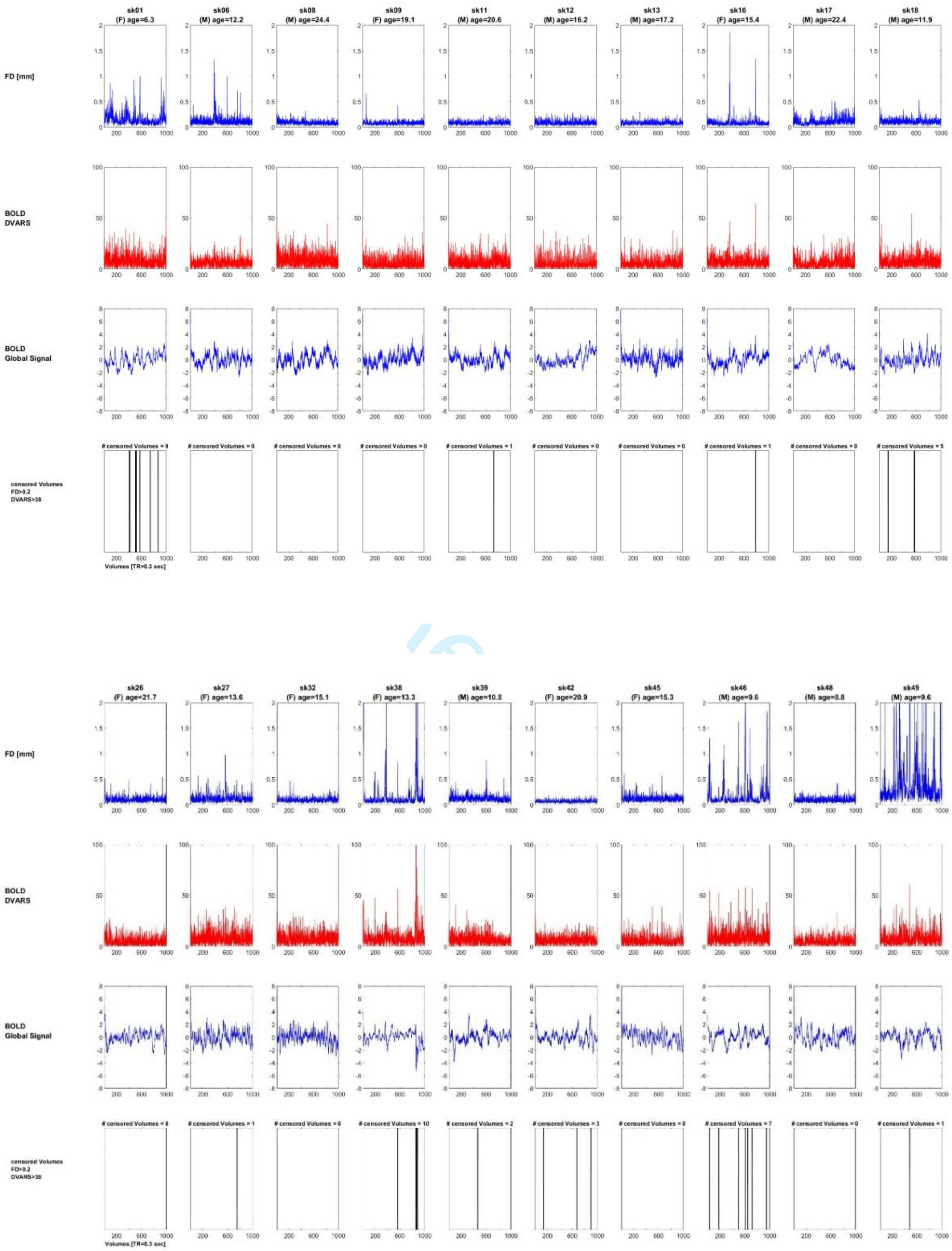

Supplementary Figure 1. Presentation of head motion during fMRI acquisition of each of the 20 patients. For each subject we display a set of 4 plots that included: 1) FD. Above this first plot we note the gender of the subject and its age; 2) DVARS; 3) BOLD global signal; 4) censured volumes. Above this plot, we note the total number of volumes that were identified with the cuts [see text above] to be censored.

Supplementary Table 2. Association between thalamo-cortical connectivity and selective attention, inhibition, and working memory. Significant clusters in different sub-nuclei of the thalamus are listed resulting from the multivariate linear regression with lesion size and age as covariates.

Network	group	Nuclei	Selective attention				Inhibition				Working Memory				P
			t(pos)	vox	t(neg)	vox.	t(pos)	vox.	t(neg)	vox.	t(pos)	vox.	t(neg)	vox.	(FDR)
Motor	ant	AV/LD	-	-	2.3-2.4	2	2.5-3.2	12	-	-	2.6-2.8	6	-	-	>0.05
	med	MD/ IL	-	-	-	-	-	-	2.5	7	2.5-2.8	8	2.4-2.5	9	
	lat	VL/VP	-	-	2.4-2.5	3	-	-	-	-	-	-	2.3-3.3	12	
	post	Pu	-	-	-	-	-	-	-	-	-	-	2.3-3.0	17	
Visual	anterior	AV/LD	-	-	-	-	2.5	5	-	-	-	-	-	-	>0.05
	medial	MD/ IL	2.4-3.0	8	2.5-3.0	12	2.3-2.7	9	-	-	-	-	2.3-2.8	62	
	lateral	VA/VL	-	-	2.9-3.5	15	-	-	2.3-4.3	19	2.3-2.9	5	-	-	
	post.	Pu/MGN	2.7-3.5	34	-	-	-	-	2.3-3.3	37	2.3-2.7	18	-	-	
Auditory	anterior	AV/LD	-	-	-	-	-	-	-	-	-	-	-	-	>0.05
	medial	MD/IL	2.5-3.0	25	-	-	-	-	-	-	2.3-3.3	12	-	-	
	lateral	VA	2.7-2.8	10	-	-	2.4-3.4	7	-	-	2.6-3.1	20	-	-	
	post.	Pu	-	-	-	-	2.3-3.4	12	-	-	-	-	-	-	
DMN	anterior	AV/LD	-	-	-	-	-	-	3.3	5	-	-	-	-	>0.05
	medial	MD/IL	-	-	-	-	-	-	3.3	5	-	-	2.4-3.1	3	
	lateral	VA	-	-	2.2-3.8	12	-	-	2.6-3.8	6	2.6-3.3	5	2.3-3.5	11	
	Post.	Pu/MGN	-	-	2.2-3.2	10	-	-	2.4-3.0	8	2.4-3.1	4	2.5-3.3	7	
DMN posterior	anterior	AV	-	-	-	-	-	-	2.7-2.8	26	-	-	2.4	2	>0.05
	medial	MD/ IL	-	-	-	-	2.3-3.2	4	-	-	2.4-4.3	3	2.8	5	
	lateral	VA/VL	-	-	-	-	2.3-2.8	8	-	-	2.3-3.0	2	2.3-3.1	12	
	post.	Pu/MGN	2.3-2.4	30	-	-	2.5-3.5	9	-	-	2.4-3.0	3	2.5-3.2	9	
Salience	anterior	AV	2.7-5.0	15	-	-	-	-	-	-	-	-	-	-	>0.05
	medial	MD/IL	2.6-3.2	8	2.5-3.0	8	2.3-2.6	8	-	-	-	-	2.4-3.2	6	
	lateral	VA/VL	-	-	2.9-3.5	10	-		-	-	-	-	2.4-3.4	9	
	post.	Pu/MGN	2.7-3.5	4	2.5-3.8	22	2.9-3.1	12	-	-	-	-	2.3-2.8	3	
Dorsal attention	anterior	AV	-	-			-	-	-	-	-	-	2.2-2.4	5	>0.05
	medial	MD/IL	-	-	-	-	-	-	-	-	-	-	2.3-2.5	7	
	lateral	VA/VL	-2.6-3.7	7	-	-	-	-	-	-	-	-	2.3-3.3	9	
	post.	Pu/MGN	-	-	-	-	-	-	-	-	-	-	2.4-2.7	7	
Left EF	anterior	AV/LD	3.0-3.2	4	-	-	-	-	-	-	-	-	-	-	>0.05
	medial	MD/IL	2.3-3.0	8	3.1	3	2.7	9	-	-	2.3-2.9	14	-	-	
	lateral	VL/VP	2.6-4.1	22	-	-	2.3-3.1	15	-	-	2.3-2.7	6	-	-	
	post.	Pu/MGN	2.2-3.4	5	-	-	2.3-3.2	17	-	-	2.4-3.3	3	-	-	
Right EF	anterior	VA	-	-	-	-	-	-	-	-	2.8-3.5	5	-	-	>0.05
	medial	MD/IL	3.10	4	-	-	2.4-2.6	3	-	-	2.8-3.5	15	-	-	
	lateral	VA	-	-	2.4-2.9	12	2.3-3.1	8	-	-	2.2-3.9	12	-	-	
	Post.	Pu/MGN	-	-	2.4-3.9	11	2.5-3.1	11	-	-	2.4-3.0	7	2.2-2.3	3	

Notes. DMN, default mode network; Dorsal Attention., dorsal attention network, Left EF, left executive network; Right EF, right executive network. AV, anteroventral; IL, intralaminar, MD, mediodorsal, Pu, pulvinar; VA, ventral anterior; VL, ventral lateral: LD, lateral dorsal; VP, ventral posterior.

For Review Only

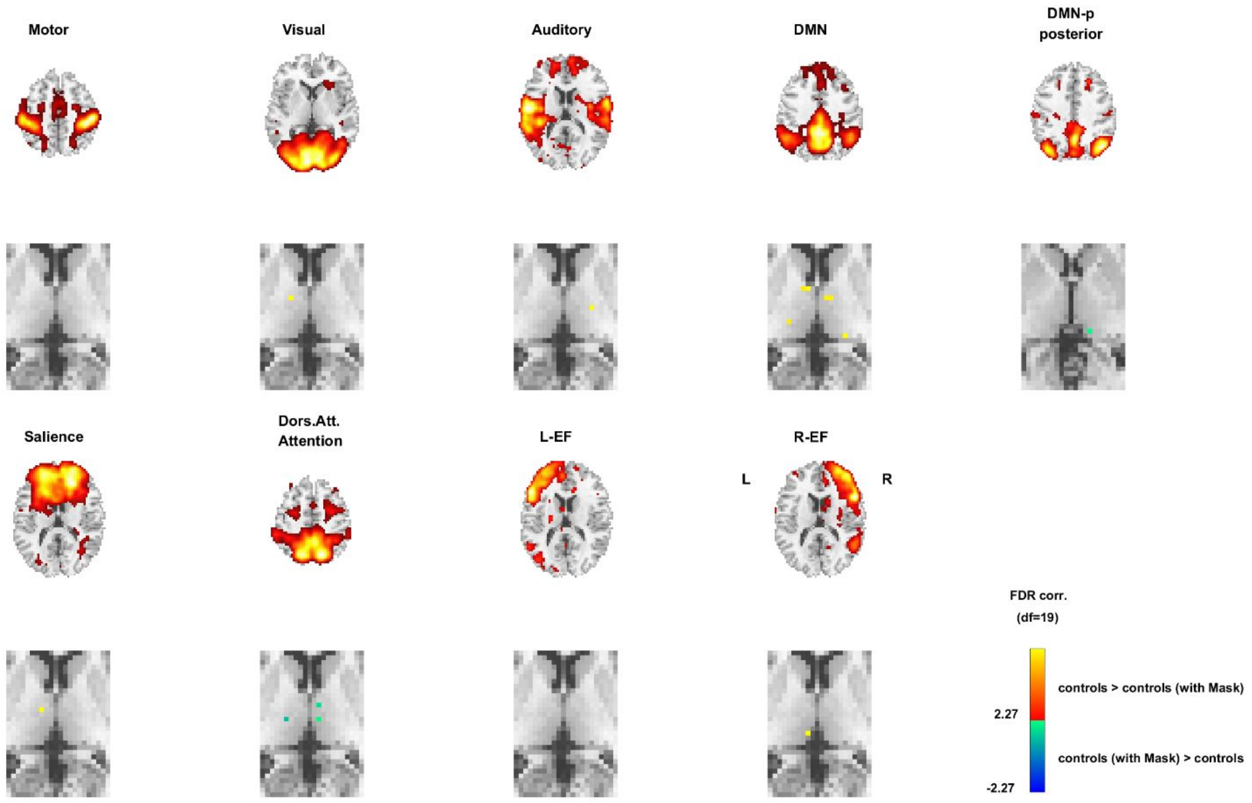

Supplementary Figure 2. Within group differences in thalamo-cortical networks. Within group test comparing healthy controls to healthy controls with the same masks that were derived from patients (e.g. the mask were exactly the same with respect to the total number of voxels and were exactly positioned at the same location as for the patients).

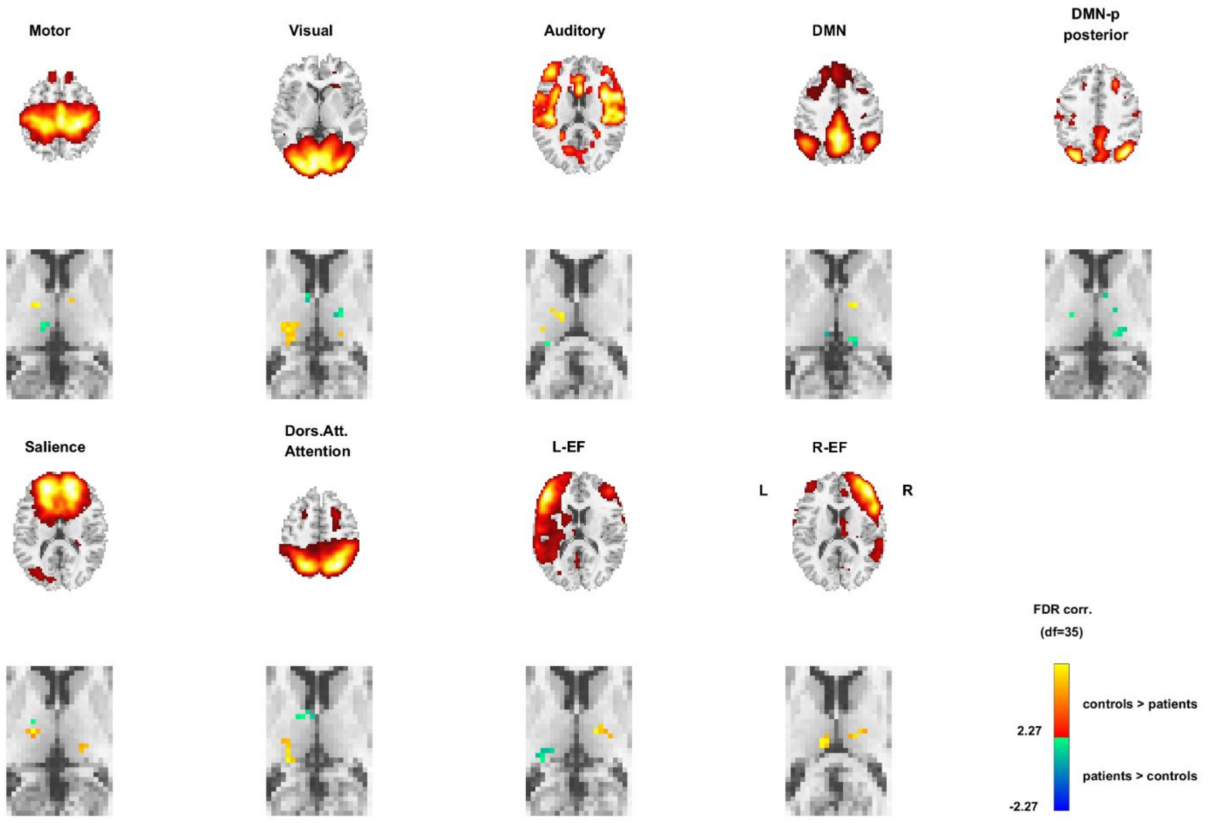

Supplementary Figure 3. Group differences in thalamo-cortical networks without patients with thalamic infarctions. As there were three patients with thalamic infarctions, we conducted a supplementary analysis investigating the thalamo-cortical networks excluding these three patients. Analyses revealed that there were only minimal differences in the t-test comparing patients and controls in the full sample vs. the sample without thalamic infarctions. Thus, these three patients did not substantially affect the results presented in the study.

References

1. Power JD, Mitra A, Laumann TO, Snyder AZ, Schlaggar BL, Petersen SE. Methods to detect, characterize, and remove motion artifact in resting state fMRI. *NeuroImage*. Jan 1 2014;84:320-41. doi:10.1016/j.neuroimage.2013.08.048
2. Power JD, Schlaggar BL, Petersen SE. Recent progress and outstanding issues in motion correction in resting state fMRI. *NeuroImage*. Jan 15 2015;105:536-51. doi:10.1016/j.neuroimage.2014.10.044
3. Steiner L, Federspiel A, Slavova N, et al. Functional topography of the thalamo-cortical system during development and its relation to cognition. *NeuroImage*. Dec 2020;223:117361. doi:10.1016/j.neuroimage.2020.117361
4. Fox MD, Zhang D, Snyder AZ, Raichle ME. The global signal and observed anticorrelated resting state brain networks. *Journal of neurophysiology*. Jun 2009;101(6):3270-83. doi:10.1152/jn.90777.2008
5. Murphy K, Birn RM, Handwerker DA, Jones TB, Bandettini PA. The impact of global signal regression on resting state correlations: are anti-correlated networks introduced? *NeuroImage*. Feb 1 2009;44(3):893-905. doi:10.1016/j.neuroimage.2008.09.036
6. Power JD, Barnes KA, Snyder AZ, Schlaggar BL, Petersen SE. Spurious but systematic correlations in functional connectivity MRI networks arise from subject motion. *NeuroImage*. Feb 1 2012;59(3):2142-54. doi:10.1016/j.neuroimage.2011.10.018
7. Smyser CD, Inder TE, Shimony JS, et al. Longitudinal analysis of neural network development in preterm infants. *Cereb Cortex*. Dec 2010;20(12):2852-62. doi:10.1093/cercor/bhq035
8. Van Dijk KR, Hedden T, Venkataraman A, Evans KC, Lazar SW, Buckner RL. Intrinsic functional connectivity as a tool for human connectomics: theory, properties, and optimization. *Journal of neurophysiology*. Jan 2010;103(1):297-321. doi:10.1152/jn.00783.2009

For Review Only

The purpose of the study was to investigate resting-state thalamo-cortical connectivity and its association with cognition in patients after pediatric arterial ischemic stroke. Steiner & Federspiel et al. report that the interaction between different subdivisions of the thalamus and several cortical networks might be essential for post-stroke cognitive performance.

For Review Only

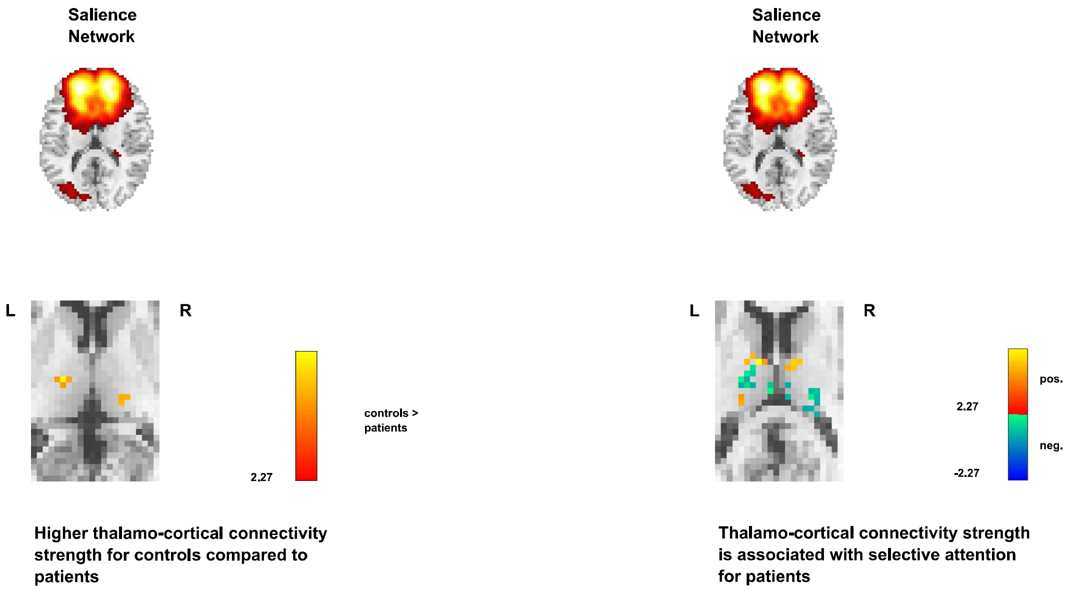
Salience Network
L
R
2.27
controls > patients
Higher thalamo-cortical connectivity strength for controls compared to patients
Salience Network
L
R
pos.
2.27
neg.
-2.27
Thalamo-cortical connectivity strength is associated with selective attention for patients
